# Karyomapping—a comprehensive means of simultaneous monogenic and cytogenetic PGD: comparison with standard approaches in real time for Marfan syndrome

**DOI:** 10.1007/s10815-014-0405-y

**Published:** 2015-01-06

**Authors:** Alan R. Thornhill, Alan H. Handyside, Christian Ottolini, Senthil A Natesan, Jon Taylor, Karen Sage, Gary Harton, Kerry Cliffe, Nabeel Affara, Michalis Konstantinidis, Dagan Wells, Darren K. Griffin

**Affiliations:** 1 School of Biosciences, University of Kent, Canterbury, CT2 7NJ UK; 2 The Bridge Centre, London, SE1 9RY UK; 3 Illumina Limited, CPC4, Capital Park, Fulbourn, Cambridge, CB21 5XE UK; 4 Department of Pathology, University of Cambridge, Tennis Court Road, Cambridge, CB2 1QP UK; 5 Reprogenetics UK, Institute of Reproductive Sciences, Oxford Business Park North, Oxford, OX4 2HW UK; 6 Nuffield Department of Obstetrics and Gynaecology, University of Oxford, Women’s Centre, John Radcliffe Hospital, Oxford, OX3 9DU UK; 7 School of Biosciences, University of Kent, Canterbury, CT2 7AF UK

## Introduction

Preimplantation genetic diagnosis (PGD) of single gene defects by genetic analysis of single or small numbers of cells biopsied from in vitro fertilization (IVF) embryos is clinically well-established. Targeted haplotyping by multiplex fluorescent polymerase chain reaction (PCR) of closely linked or intragenic short tandem repeat (STR) markers combined with direct mutation detection improves the accuracy of single cell analysis significantly and minimizes potential errors caused by undetected allele dropout (ADO) or contamination [1]. Allele dropout refers to the failure of one of the two alleles of a heterozygous locus to amplify. This makes a heterozygous cell appear homozygous at the affected locus, potentially leading to misdiagnosis. Furthermore, using high order multiplex protocols, this approach has been extended to multiple loci, including analysis of the Human leukocyte antigen (HLA) region for selection of embryos tissue matched to existing sick children and diagnosis of translocation chromosome imbalance [2–4]. However, the development of patient, disease or locus-specific protocols, and testing with single cells, is time-consuming and labour intensive. Also, this targeted approach only provides limited information on chromosome aneuploidy, which is recognized to be a major cause of IVF failure and pregnancy loss.

As an alternative, we developed, “Karyomapping”—genome wide parental haplotyping using high density single nucleotide polymorphism (SNP) genotyping. Karyomapping provides a comprehensive method for linkage-based diagnosis of any single gene defect [5]. Genotyping of the parents and a close relative of known disease status, to phase informative SNP loci, eliminates the need for customized test development and, as Karyomapping defines four sets of SNP markers for each of the parental chromosomes, it allows simultaneous high-resolution molecular cytogenetic analysis. Thus, meiotic trisomies, including their parental origin, can be identified by the presence of both haplotypes from one parent in segments of the chromosome, resulting from the inheritance of two chromosomes with different patterns of recombination. Moreover, monosomies or deletions can be identified by the absence of either chromosome haplotype from the parent of origin [5]. Mitotic chromosome duplication, which can arise through malsegregation of chromosomes in the cleavage divisions following fertilization, cannot be detected by Karyomapping per se, since the sequence of both chromosomes is identical. However, chromosome duplications may be clinically less significant, since they are often associated with poor morphology and developmental arrest.

In the past we have demonstrated that Karyomapping could be used for the detection of cystic fibrosis status in single cells [5]. Here we provide proof of principle for the widespread clinical application of Karyomapping, first by adapting the protocol for clinical use in a regular PGD timeframe (24 h) and secondly by detection of the autosomal dominant condition Marfan syndrome. Performing Karyomapping “as if in a clinical setting” for confirmation of results of an existing PGD case provides strong evidence of the applicability of Karyomapping and, in this case, led to a twin birth.

## Materials and methods

### Patient history

Marfan syndrome is an autosomal dominant disorder of the connective tissue predisposing to aortic aneurism and caused by mutations in the fibrillin-1 (*FBN1*) gene on chromosome 15q21.1. A couple, in which the father is affected by Marfan syndrome and has had an aorta replacement and treatment for a detached retina, requested preimplantation genetic diagnosis (PGD). The father was previously referred to us by an accredited National Health Service (NHS) laboratory as heterozygous for two mutations in *FBN1*, c.235C>T and c.3089A>G. The first, c.235C>T (p.Gln79X) is a nonsense change that has been reported in the FBN1 online mutation database http://www.umd.be/FBN1/4DACTION/WV/2699. The second variant is a missense change c.3089A>G (p.Asn1030Ser), this was not reported in the database at the time of writing. While the database does not assign specific pathologies to each mutation, the reasonable assumption was made that one or both of these mutations in *FBN1* were the cause of Marfan Syndrome in this patient. While there was no molecular work up of older family members there was also no prior family history of the syndrome. Both were found to be present in his affected daughter (5 years old at the time of treatment) establishing that they are present in *cis* on the same paternal chromosome. The mother (36 years old at the time of treatment) had only one other natural pregnancy that resulted in a hydatidiform mole.

### IVF cycle

An antagonist protocol was used for ovarian stimulation. When the average follicular diameter was >16 mm, 5000 IU β-human Chorionic Gonadotrophin (β-hCG) was administered and the oocytes retrieved 36 h later by ultrasound-guided transvaginal aspiration under local anaesthesia. Intracytoplasmic sperm injection (ICSI) was used for insemination of mature oocytes, 6–8 h after the oocyte retrieval, to avoid contamination by extraneous sperm. The following morning (Day 1), each injected oocyte was checked for pronuclei to confirm fertilization.

### Embryo biopsy

Normally fertilized embryos (with two pronuclei on Day 1), which developed to the 6- to 10-cell stages on Day 3 following ICSI were transferred to calcium- and magnesium-free medium (Quinn’s Advantage, Cooper Surgical, CT, USA) and one or two single blastomeres were biopsied for genetic analysis by micromanipulation after making an opening in the zona pellucida using a non-contact infrared laser (Saturn 3, Research Instruments Ltd, Penryn, UK). The embryos were then returned to culture while the biopsied cells were thoroughly washed in non-stick wash buffer [phosphate buffered saline (PBS) with 0.1 % polyvinyl pyrrolidone]. The washed cells were transferred to 0.2 ml PCR tubes in approximately 1–2 μl of the wash buffer and frozen before transportation to Reprogenetics UK (Oxford, UK). The whole genome of each single cell was amplified by multiple displacement amplification (MDA). For the clinical diagnosis, targeted haplotyping and direct mutation detection of the MDA products was used.

### Whole genome amplification

The whole genome of the single blastomeres was amplified by multiple displacement amplification (MDA) [6] according to the manufacturer’s instructions with modifications (Repli-g Midi kit, Qiagen, Germany). In brief, 1.5 μL of PCR-grade water were added to each sample and alkaline lysis carried out by adding 2.5 μl of lysis buffer (0.75 μL of PCR-grade water, 1.25 μL of 0.1 M DTT and 0.5 μL of 1 M NaOH) and incubation at 60 °C for 10 min. Neutralization buffer (2.5 μl 0.4 M Tricine), 12.5 μl PCR grade water, 29 μl reaction buffer and, finally, 1 μl of DNA polymerase (Repli-g Midi kit, Qiagen, Germany) was added to each sample individually for a final reaction volume of 50 μl. The samples were then incubated in a thermocycler at 30 °C for 2 h, followed by enzyme inactivation at 65 °C for 5 min.

### Short tandem repeat (STR) and mutation analysis

For dominant conditions, PGD protocols that focus on the analysis of the mutation site alone are associated with an unacceptably high risk of misdiagnosis caused by ADO. Allele dropout is common at the single cell level and, as explained above, results in a heterozygous cell appearing to be homozygous. In the case of PGD for Marfan syndrome, ADO affecting the mutation site on the copy of chromosome 15 carrying the mutation, could cause an affected embryo appear normal (as only the normal allele is successfully amplified). To reduce the risk of misdiagnosis, a strategy employing a combination of mutation detection and analysis of closely linked short tandem repeat (STRs) was used [7–9], revealing the paternal 15q21.1 haplotype associated with the mutation. Only one STR marker (D15S659) was found that had different repeat alleles on each of the four parental chromosomes and was fully informative (Table 1). To increase accuracy, a further two STRs, one intragenic (D15S196) and a second proximal STR (D15S143), were selected, which had two paternal alleles one of which was shared with the single maternal allele.

**Table 1:** STR sizes for family members (see also Fig. 1)

Patients tested	Short tandem repeats		
	D15S143^a^ (Proximal flanking)	D15S196^a^ (Intragenic)	D15S659^a^ (Proximal)
Father (affected carrier)	**185** / 193	**271** / 275	**176** / 180
Mother (unaffected)	193	275	192 / 200
Daughter (affected carrier)	**185** / 193	**271** / 275	**176** / 200

^a^Linked marker alleles in **bold** are the ones associated with the **mutant** *FBN1* allele

Following isothermal MDA, the products were amplified in a series of singleplex PCR reactions. Reaction mixtures contained PCR grade water (Roche, Germany), 1x HotMaster Taq Buffer (with 25 mM Mg2+) (5 Prime, Germany), dNTPs (200 μM each) (Thermo Scientific, USA), 0.8 μM each primer, 0.6 units HotMaster Taq DNA polymerase and 1 μl MDA WGA DNA for a final volume of 15 μl. Thermal cycling consisted of an initial denaturation step of 96 °C for 1 min, followed by 50 cycles of 94 °C for 15 s, 60 °C for 15 s, and 65 °C for 45 s, then a final extension step of 65 °C for 2 min. Primer sequences for the linked STRs were obtained from the NCBI ‘UniSTS’ database (http://www.ncbi.nlm.nih.gov/unists): D15S1435′-CCTAAGGAGGCAACAGCAAAG-3′ and 5′-GTAAAGACTGGTATCTGTAGCAC-3′); D15S196 (5′-GACCTGTAGCTGAAGGGAAG-3′ and 5′- ATAAAAGTGGTGGGGAAGGATG-3′);D15S659 (5′- GTGGATAGACACATGACAGATAGG-3′ and 5′- TATTTGGCAAGGATAGATACAGG-3′). The primers utilised for amplification of the mutation site were: 5′- TGGATGGAAAACCTTACCTG-3′ and 5′- CAGTTACAAAAGGCCACATTC-3′. Only the c.235C>T mutation was targeted since the two mutations identified in father and daughter were determined to be in *cis* (i.e. located within the same gene on the same chromosome and therefore inherited together).

### Mutation detection

The procedure for carrying out minisequencing involved two separate reactions. Initially, products derived from PCR amplification of the mutation site were treated with ExoSAP-IT (USB, Affymetrix, USA) according to manufacturer’s instructions. Treated products were then subjected to minisequencing through the usage of the SNaPshot Multiplex Kit (Applied Biosystems, USA). Specifically, the reaction mixture contained 2.5 μl of SNaPshot Multiplex Ready Reaction Mix, 0.5 μl of 2 μM minisequencing primer (F–5′-AAACCTTACCTGGCGGAAAT-3′), 0.5 μl PCR grade water and 1.5 μl of treated amplified product for a final volume of 5 μl. Thermal cycling consisted of 25 cycles of 96 °C for 10 s, 50 °C for 5 s, 60 °C for 30 s.

### Single nucleotide polymorphism genotyping and Karyomapping

For Karyomapping validation, genomic DNA from both parents and their affected daughter and the same MDA products were used for SNP genotyping at approximately 300,000 loci genome wide using a short 24 h protocol. During early work up of the protocol we optimised the array with well-distributed snps with high heterozygosity indeces. All DNA samples (including controls and MDA products from single blastomeres) were subjected to SNP genotyping on Human CytoSNP-12 bead array according to the manufacturer’s instructions (Illumina, Inc.) using either the conventional 72 h protocol or a shortened 24 h protocol designed for clinical application in a PGD cycle. Processed arrays were scanned and the image data analysed and converted to genotype data (GenomeStudio;Illumina, Inc). By processing the parents and the reference and analysing them using Karyomapping, we can identify the number of informative SNPs for that particular family (both paternal and maternal informative SNPs). The genotype data was finally exported as an Excel compatible file for Karyomap analysis. In this study Visual Basic for Applications (VBA) macro in Microsoft Excel was used to analyse the SNP genotype data and display Karyomaps as previously described [5]. The Karyomaps of each embryo were then analyzed blind for concordance with the original diagnosis and any chromosome aneuploidy. The consistency of Karyomapping at the single cell level was investigated by follow up analysis of the whole biopsied embryo and in one affected embryo, multiple single cells, following disaggregation. Full details of the Karyomapping protocol have been recently published [10, 11]—these deal with the shortened protocol, the validation of the SNP chip approach plus the independent corroboration of cytogenetic data using array CGH. Recent unpublished data with parallel array CGH and Karyomapping continues to confirm all monosomies, deletions and meiotic trisomies.

### Follow up analysis of embryos

Three embryos identified as having chromosomal abnormalities by Karyomapping were cultured to Day 6 post-ICSI. The zona pellucida was removed from each embryo before washing and transferring whole embryos into PCR tubes in 2 μl PBS. The single affected embryo was disaggregated to single cells in calcium- and magnesium-free medium on Day 4 post-ICSI and the cells individually washed and transferred to PCR tubes. All samples were stored at −20 °C. After thawing the whole genome was amplified by MDA and the products genotyped and analyzed as described above.

## Results

### IVF and embryo biopsy

Ten cumulus oocyte complexes were collected and eight mature oocytes, arrested at metaphase II, inseminated by ICSI. The following morning the injected oocytes were checked and six had two pronuclei indicating normal fertilisation. All six embryos reached appropriate cleavage stages between the 6- to 10-cell stages on Day 3 post ICSI and one or two cells were biopsied for genetic analysis.

### Targeted haplotyping and direct mutation analysis

**Fig. 1:**
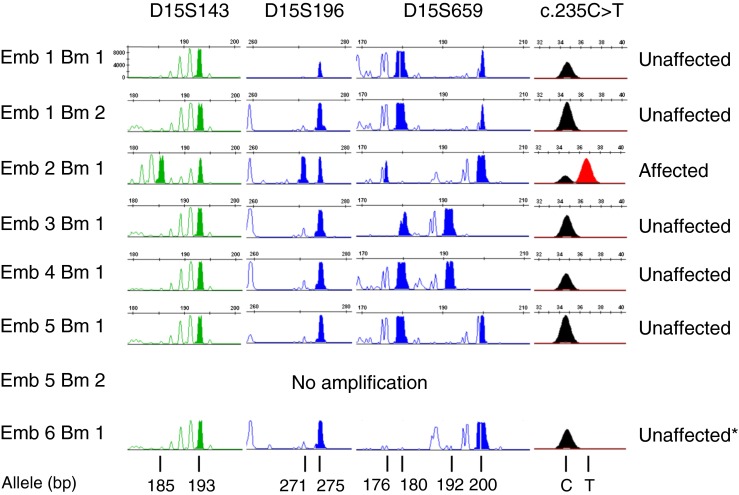
Analysis of three short tandem repeat (STR) markers and thec.235C>T mutation in *FBN1* by capillary electrophoresis

Following whole genome amplification, targeted haplotyping, with all three STR markers, and direct mutation analysis was successful in 7/8 (87.5 %) of the single cells biopsied from six cleavage stage embryos (Table 2; Fig. 1). Two single cells were biopsied from two embryos but one of these from Embryo 5 failed to amplify. Analysis of the STR alleles present at the *FBN 1* locus were consistent with the mutation status in five embryos and identified four as unaffected (Embryos 1, 3–5) and one as affected (Embryo 2). In the remaining single cell biopsied from Embryo 6 with a normal allele for the mutation, only one of the maternal alleles (200 bp) and neither of the paternal specific alleles (176 and 180 bp) were detected with D15S659. Furthermore, the other two STR markers had only a single allele shared by both parents (193 and 275 bp, respectively). The interpretation was therefore that either the paternal chromosome with the normal *FBN1* allele was present but that allele dropout (ADO) had occurred with D15S659, or the paternal chromosome 15 was absent from that cell and was reported as unaffected with reduced accuracy.

**Table 2:** Comparison of targeted haplotyping, direct mutation analysis with Karyomapping for linkage based diagnosis of Marfan syndrome and cytogenetic analysis with Karyomapping in single cells biopsied from cleavage stage embryos

Embryo (blasto -mere)	Short tandem repeats (bp)			Mutation (c.235C>T)	Interpretation	Karyomap analysis	? Concordant	Paternal Chromosome		Maternal Chromosome		Comment
	Paternal/Maternal					Paternal /Maternal		Gain	Loss	Gain	Loss	
1 (1)	193	275	180/200	Normal allele only	Unaffected^c^	P2/M1	Yes				21	Mosaic loss of chr21
1 (2)	193	275	180/200	Normal allele only	Unaffected^c^	P2/M1	Yes					
2	**185**/193^a^	**271**/275^a^	**176**/200^a^	Normal & mutant allele	Affected	P1/M1	Yes			1 (MeII)		
3	193	275	−/192^b^	Normal allele only	Unaffected^c^	-/M2 (Mat genome only)	Yes^b^		All chrs	10 (MeI)	19	No paternal genome
4	193	275	180/192	Normal allele only	Unaffected	P2/M2	Yes		6qter^d^			**Embryo Transferred**
5 (1)	193	275	180/200	Normal allele only	Unaffected	P2/M1	Yes					**Embryo Transferred**
5 (2)	NR	NR	NR	Normal allele only	Amplification failure	NR	N/A					No amp
6	193	275	200	Normal allele only	Unaffected^c^ (reduced accuracy)	−/M1	Yes, (given cytogenetic result)		15, 20 qter	6p dup, 8 (MeII)		Paternal monosomy 15

**For chromosomal analysis:** *MeI/MeII* meiosis I/II, *qter* terminal portion of long arm of the chromosome, *dup* duplication, *all chrs* all chromosomes

**For Karyomap analysis:** *P1* paternal haplotype 1, *P2* paternal haplotype 2, *M1* maternal haplotype 1; *M2* maternal haplotype 2

^a^Linked marker alleles in **bold** are the ones associated with the **mutant** *FBN1* allele

^b^Note an allele was originally determined to be present at 180 bp but, after subsequent analysis, determined to be an artefact

^c^Although determined as not carrying the mutant Marfan allele, embryo not transferred for other (e.g. cytogenetic) reasons

^d^This apparent deletion was identified after retrospective closer analysis of Karyomapping traces. As call rates were low in this region, it is possible that the apparent deletion was a technical artefact

### Karyomapping analysis

**Fig. 2:**
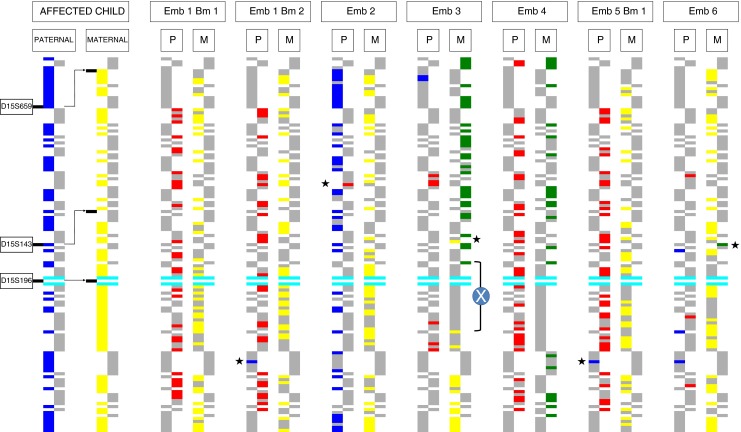
Detailed Karyomaps for chromosome 15q21.1 in single blastomeres biopsied from each cleavage stage embryo. Consecutive informative single nucleotide polymorphism (SNP) loci for the four parental chromosomes are represented by two pairs of columns in each case (paternal, left and maternal, right) in which each segment is an informative SNP. Single cell genotypes identifying the presence of one of the four parental chromosomes at informative SNP loci are coloured (paternal chromosomes P1 and P2 are indicated in *blue* and *red* respectively; maternal chromosomes M1 and M2 in *yellow* and *green* respectively). The Karyomaps of a 5–6 Mb region of chromosome 15q21.1 of the affected child, known to be a carrier of both paternal mutations (P1—*blue*) and used as a reference for phase, and seven single blastomeres biopsied from six cleavage stage embryos are presented (M1—*yellow* chromosome also assigned). Otherwise, informative SNP genotypes, which indicate the absence of that chromosome or are not called, are coloured *grey*. The position of fibrillin-1 (*FBN1*) relative to the SNP loci is indicated by the *light blue bars*. The positions of the three short tandem repeat (STR) markers, D15S143, D15S196 and D15S659 used for conventional analysis are indicated on the left. Three embryos are identified as having the unaffected (*red*) paternal chromosome (Embryos 1, 4 and 5); one embryo has the affected paternal chromosome (*blue*) also present in the affected child (Embryo 2), and two embryos are missing the paternal chromosomes either because of the complete absence of the paternal genome in a parthenogenetically activated embryo (Embryo 3) or paternal monosomy 15 (Embryo 6). (See “Results” and “Discussion” Sections for more detail). The genomic DNA from the affected child, the first single blastomere from Embryo 1 and the two abnormal embryos, Embryos 3 and 6, were genotyped on the normal 3 day protocol. For comparison, all of the other cells, including the second blastomere from Embryo 1, were genotyped using the 24 h protocol. Abbreviations used: *Emb* embryo, *Bm* blastomere (cell), *X* crossover, asterix* miscall

SNP genotyping and Karyomaping analysis was successful with all seven single cells from which MDA products were available. Genomic DNA samples from the parents and affected child gave call rates of about 97 % and heterozygous call rates of 28–29 %. Overall SNP call rates were lower in si ngle cells, ranging from 78 to 82 % with a significant rate of ADO (approximately 15 %) and heterozygous call rates ranging from 14 to 20 % (excluding the cell from the parthenogenetic haploid embryo). Karyomapping identified the parental chromosomes present at the *FBN1* locus on chromosome 15q21.1 in 11/12 (92 %) of the chromosomes present in the seven cells analyzed (Fig. 2, Table 2). The only chromosome which could not be haplotyped confidently at the *FBN1* locus was the maternal chromosome 15 in Embryo 3. In that case, there was a crossover immediately distal to *FBN1* and without any intragenic informative SNP loci, the exact location of the recombination event could not be identified unequivocally. Otherwise, the paternal and maternal haplotypes identified (where present) were concordant with the targeted haplotyping and direct mutation analysis, including the proximal region of the maternal chromosome 15 in Embryo 3 (Table 2).

In two embryos, Karyomapping revealed that the paternal chromosome 15 was absent with only a low proportion of random positive informative SNPs for both haplotypes (Embryos 3 and 6; Fig. 2). For Embryo 3, examination of the Karyomaps for all the other chromosomes revealed that no paternal chromosomes were present, which may have resulted from fertilization failure and parthenogenetic activation of development, whereas in Embryo 6, the missing chromosome was an isolated paternal monosomy (Table 2).

Karyomapping analysis of the other chromosomes present in the single cells revealed several aneuploidies and structural abnormalities (Table 2). These included three maternal meiotic trisomies in three separate embryos: trisomy 1 in Embryo 2 in which both maternal chromosome haplotypes were detected on segments of both arms of the chromosome (meiosis II type), trisomy 10 in Embryo 3 with both maternal haplotypes additionally present across the centromere (meiosis I type) and trisomy 8 (meiosis II type) in Embryo 6. In addition, there was a mosaic loss of maternal chromosome 21 in Embryo 1, and possible deletions affecting paternal 6q and 20q in Embryos 4 and 6, respectively. The deletion for chromosome 6q in embryo 4 was discovered after a more detailed retrospective analysis and a low call rate in that region for this blastomere meant that we were not 100 % confident with this diagnosis. Finally, both maternal chromosome haplotypes were present for a segment of the short arm only for chromosome 6 in Embryo 6 (partial trisomy 6p). As this abnormality was not confirmed in the whole embryo (see below), this may be an acentric fragment from the other maternal chromosome, which arose by chromosome breakage during meiosis, and remained in the oocyte at fertilization.

### Follow up analysis of embryos

Analysis of the three embryos that were not selected for transfer and were processed whole, confirmed the presence of the two meiotic trisomies and the maternal loss of chromosome 19 in Embryos 3 and 6 (Table 2). However, as expected the mosaic loss of maternal chromosome 21 in Embryo 1 was not detected. Furthermore, the presence of both maternal chromosomes for 6p in Embryo 6 was also not detected. This is consistent with the presence of an acentric fragment arising in meiosis and segregating to the biopsied blastomere since an extra whole chromosome would normally be present in most, if not all, cells as was observed for the other meiotic trisomies.

A fourth embryo (Embryo 2) was disaggregated on Day 4 into 8 single blastomeres and one two-cell sample (10 cells in total). All of these cells had identical Karyomaps and the presence of trisomy 1 was confirmed in each case (Table 2). There were no other mosaic chromosome abnormalities except for partial loss of maternal chromosome 13 (approximately 40.5 Mb) in one cell.

### Clinical outcome

Based on the results of the targeted haplotype and direct mutation analysis (see above), two embryos diagnosed as unaffected were transferred resulting in a twin pregnancy. Delivery was premature at 28 weeks and subsequently one of the twins died following a perinatal infection. The remaining twin boy was healthy at 2 years. Another of the unaffected embryos (Embryo 1; Table 2), cryopreserved by vitrification at the blastocyst stage on Day 6 post ICSI, was successfully thawed 16 months later and transferred in an unstimulated cycle; no pregnancy resulted.

## Discussion

The results of this study demonstrate the clinical utility of a novel, comprehensive approach for PGD (Karyomapping) that combines detection of any monogenic disorder (potentially) with comprehensive chromosome screening in a single test that requires no a priori development. Comparison with a well-established strategy (minisequencing for direct mutation detection combined with linked STR marker analysis) suggests that Karyomapping, could be applied clinically for the autosomal dominant condition, Marfan syndrome. Specifically after whole genome amplification of single blastomeres biopsied from cleavage stage embryos, both methods (performed in parallel) identified unaffected and affected embryos with high efficiency and accuracy. Karyomapping however had the added advantage of not requiring the clinical work-up of a specific test beforehand (only the SNP array information from the parents and an affected child was needed).

Minisequencing in combination with the analysis of several STR markers [11–14], yielded results within 24 h for all six embryos in which whole genome amplification of the single cells was successful. Furthermore, in all cases, each locus was successfully re-amplified from the MDA products with no detectable allele dropout (ADO). Because however STR analysis in this way is not quantitative, and two of the markers were only semi-informative, ADO cannot be completely excluded in the unaffected embryos (Fig. 1). In the affected embryo, single paternal and maternal repeat alleles were detected for all three STR markers and both the normal and mutant alleles were detected by minisequencing. Although this would, in practice, lead to a low probability of misdiagnosis, the mere presence of ADO at one or two loci might nonetheless undermine confidence in the result. Indeed, one result (Embryo 6; Table 2) was reported to be of lower accuracy because no paternal specific repeat alleles were detected. Karyomapping following SNP genotyping on the other hand identified 122 informative SNP loci across each of the two paternal and two maternal chromosomes in an approximately 5 and 6 Mb region spanning the *FBN1* locus on chromosome 15q21.1 (Fig. 2). Even taking into account the significant incidence of ‘no calls’ at heterozygous loci, the density of positive informative SNPs for the chromosome present, together with the absence of positive informative markers for the other chromosome, delivered highly accurate haplotyping—significantly more accurate than haplotyping with individual loci. Conservatively, the accuracy of Karyomapping could be calculated as the probability of a double recombination between the nearest flanking positive (or negative) informative SNP loci. For the single cells in this family, this ranged from about 0.5 to 1 Mb across *FBN1*. Thus the probability of a double crossover in this region would be less than 0.25–0.5 × 10^−4^ assuming 1 % recombination per megabase [13].

**Fig. 3:**
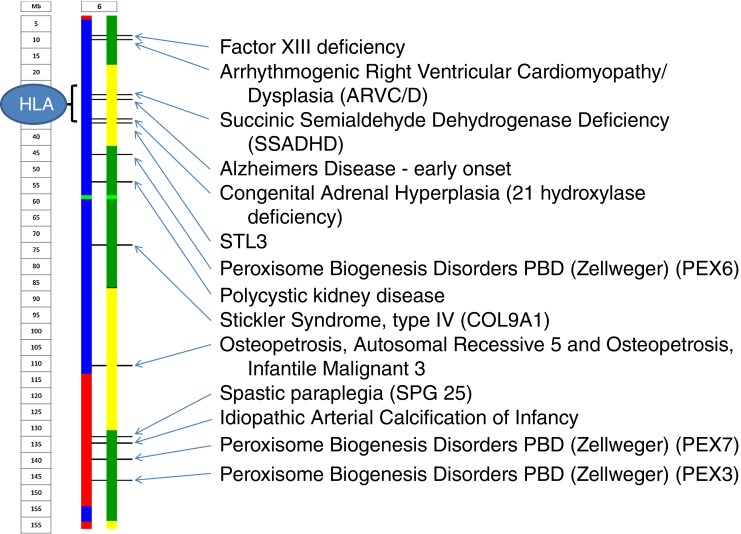
Karyomap of a single blastomere focussing on a region of chromosome 6. The image indicates that there are 11 loci in this region corresponding to disorders currently licensed for PGD by the HFEA. This includes the HLA regions used for diagnoses of saviour siblings

Figure 3 illustrates also the flexibility and utility of Karyomapping in that it can in theory be used for multiple loci simultaneously. For instance there are 11 loci corresponding to disorders licensed for PGD by the HFEA (Human Fertilization and Embryology Authority) in the region captured in this figure including the HLA regions which is used for diagnoses involving so called “saviour siblings.”

The primary purpose of PGD cycles is to identify embryos with a high probability of transmitting a genetic disorder. The inclusion of comprehensive chromosome screening, in addition to diagnosis of a familial mutation, is a powerful combination, especially when applied in a single assay. Given that chromosomal errors are common in embryos, leading to high rates of embryo implantation failure, miscarriage and, more birth defects, there is a sound basis for including comprehensive chromosome analysis alongside PGD. Furthermore, the additional information relating to parental origin of meiotic errors provided by Karyomapping (but not derivable from array comparative genomic hybridization data) should be particularly helpful for couples in determining which therapeutic intervention to try next (e.g. donor oocytes or donor sperm). In theory, screening for spontaneously arising aneuploidies should increase the likelihood that the embryo chosen for transfer will establish a viable pregnancy and ultimately a healthy child. Indeed, as a selection tool, aneuploidy screening can prioritize the embryo for transfer to achieve improved implantation rates and lower miscarriage rates in fresh transfer cycles [14] as well as support single embryo transfer policy as part of the drive towards reducing multiple birth rates [15].

In the current study, the benefits of Karyomapping over, for instance, more established molecular cytogenetic approaches [e.g. array comparative genomic hybridization (array CGH)] were immediately apparent. As an example, maternal meiotic trisomy of chromosome 1 was detected in a single cell of embryo 2 then subsequently detected in all other cells of that embryo. Meiotic errors are more likely to lead to clinical problems as they are more likely to affect all or most of the cells in the embryo whereas post-zygotic errors might affect fewer cells depending on the cleavage division in which they occur [16]. For instance the detection of monosomy 21 in embryo 1 was clearly a post-zygotic one that affected one cell and not the rest of the embryo. The added advantage of knowing the meiotic origin of aneuploidy may thus further improve success rates of PGD following aneuploidy screening when compared to other techniques including array CGH and quantitative fluorescent PCR. The detection of structural chromosome imbalance is also currently an advantage of Karyomapping compared to state of the art use of comparative genomic hybridization (which struggles to detect small abnormalities when using DNA amplified from single cells (e.g. [17]). Four structural abnormalities and their origins were clearly identified in this study, with a fifth possibly occurring in a single cell of an embryo that was transferred. In this case, the possibility of a deletion was only identified after a retrospective analysis of the Karyomapping data and thus, at present, Karyomapping is not validated for microdeletions. Unlike the other four abnormalities discovered therefore we cannot be certain whether the result was real or artifact. Nonetheless the absence of any congenital abnormality associated with a terminal 6q deletion in either the surviving twin or the one that perished suggests that it was not a chromosome abnormality present in the majority of the embryo. Finally, Karyomapping determined that embryo 3 had no paternal genome and presumably arose as a result of a parthenogenetically activated diploid oocyte. Such conceptuses lead to ovarian teratomas not consistent with ongoing pregnancy; notably array comparative genomic hybridization would have diagnosed such a conceptus as normal.

With each application of new technology there is an inevitable ethical debate particularly in the area of assisted reproduction. Fortunately, the law regarding PGD in the UK is very clear. The indication for performing the procedure of embryo biopsy as a precursor to diagnostic testing must meet a series of legal tests. These include the seriousness of the disorder and the likelihood that any child born would suffer from it. In addition, the condition itself for which any preimplantation test is applied must be licensed (in the UK, this would be by the Human Fertlization and Embryology Authority (HFEA)). However, there are several additional concerns raised by the ability to obtain large amounts of genetic information from the entire genome [18]. One frequently raised ethical concern relating to the ability to screen an ever-increasing number of genes simultaneously is the notion of designing a baby. However, the probability of selecting an “ideal” embryo from a typical cohort of approximately 10–15 embryos is vanishingly small even when considering only five loci. Of more pragmatic concern is the very real possibility of incidental findings with unknown significance as evidenced in this report for chromosomal disorders. The detection of hitherto unreported copy number variants as well as small or partial deletions and duplications of unknown pathological significance underlines the importance of and need for comprehensive genetic counseling throughout the PGD process.

## References

[1] Harton GL, De Rycke M, Fiorentino F, Moutou C, SenGupta S, Traeger-Synodinos J (2011). ESHRE PGD consortium best practice guidelines for amplification-based PGD. Hum Reprod.

[2] Handyside AH, Xu K (2012). Preimplantation genetic diagnosis comes of age. Semin Reprod Med.

[3] Renwick P, Trussler J, Lashwood A, Braude P, Ogilvie CM (2010). Preimplantation genetic haplotyping: 127 diagnostic cycles demonstrating a robust, efficient alternative to direct mutation testing on single cells. Reprod BioMed Online.

[4] Verlinsky Y, Rechitsky S, Schoolcraft W, Strom C, Kuliev A (2001). Preimplantation diagnosis for Fanconi anemia combined with HLA matching. JAMA.

[5] Handyside AH, Harton GL, Mariani B, Thornhill AR, Affara N, Shaw M-A (2010). Karyomapping: a universal method for genome wide analysis of genetic disease based on mapping crossovers between parental haplotypes. J Med Genet.

[6] Handyside AH, Robinson MD, Simpson RJ, Omar MB, Shaw MA, Grudzinskas JG (2004). Isothermal whole genome amplification from single and small numbers of cells: a new era for preimplantation genetic diagnosis of inherited disease. Mol Hum Reprod.

[7] Dreesen JC, Jacobs LJ, Bras M, Herbergs J, Dumoulin JC, Geraedts JPM (2000). Multiplex PCR of polymorphic markers flanking the CFTR gene; a general approach for preimplantation genetic diagnosis of cystic fibrosis. Mol Hum Reprod.

[8] Fiorentino F, Biricik A, Nuccitelli A, De Palma R, Kahraman S, Iacobelli M (2006). Strategies and clinical outcome of 250 cycles of Preimplantation Genetic Diagnosis for single gene disorders. Hum Reprod.

[9] Fiorentino F, Kahraman S, Karadayi H, Biricik A, Sertyel S, Karlikaya G (2005). Short tandem repeats haplotyping of the HLA region in preimplantation HLA matching. Eur J Hum Genet.

[10] Natesan SA, Coskun S, Qubbaj W, Prates R, Munne S, Coonen E (2014). Genome wide Karyomapping accurately identifies the inheritance of genetic defects in human preimplantation embryos in vitro. Genet Med.

[11] Natesan SA, Handyside AH, Thornhill AR, Ottolini CS, Sage K, Summers MC (2014). Live birth after PGD with confirmation by a comprehensive approach (Karyomapping) for simultaneous detection of monogenic and chromosomal disorders. Reprod BioMed Online.

[12] Fiorentino F, Kokkali G, Biricik A, Stavrou D, Ismailoglu B, De Palma R (2010). Polymerase chain reaction-based detection of chromosomal imbalances on embryos: the evolution of preimplantation genetic diagnosis for chromosomal translocations. Fertil Steril.

[13] Broman KW, Weber JL (2000). Characterization of human crossover interference. Am J Hum Genet.

[14] Scott RT, Upham KM, Forman EJ, Hong KH, Scott KL, Taylor D (2013). Blastocyst biopsy with comprehensive chromosome screening and fresh embryo transfer significantly increases in vitro fertilization implantation and delivery rates: a randomized controlled trial. Fertil Steril.

[15] Yang Z, Liu J, Collins GS, Salem SA, Liu X, Lyle SS (2012). Selection of single blastocysts for fresh transfer via standard morphology assessment alone and with array CGH for good prognosis IVF patients: results from a randomized pilot study. Mol Cytogenet.

[16] Fragouli E, Alfarawati S, Spath K, Jaroudi S, Sarasa J, Enciso M, et al. The origin and impact of embryonic aneuploidy. Hum Genet. 2013;132(9):1001–13. 10.1007/s00439-013-1309-0 23620267

[17] Vanneste E, Voet T, Caignec C, Ampe M, Konings P, Melotte C (2009). Chromosome instability is common in human cleavage-stage embryos. Nat Med.

[18] Hens K, Dondorp W, Handyside AH, Harper J, Newson AJ, Pennings G (2013). Dynamics and ethics of comprehensive preimplantation genetic testing: a review of the challenges. Hum Reprod Update.

